# Graduate medical education scholarly activities initiatives: a systematic review and meta-analysis

**DOI:** 10.1186/s12909-018-1407-8

**Published:** 2018-12-22

**Authors:** William Wood, Jonathan McCollum, Promil Kukreja, Imelda L. Vetter, Charity J. Morgan, Ana Hossein Zadeh Maleki, Lee Ann Riesenberg

**Affiliations:** 1 Affiliated Anesthesiologists, Oklahoma City, OK USA; 2 Department of Medicine, Indiana University, Indianapolis, IN USA; 3 Department of Anesthesiology and Perioperative Medicine, University of Alabama at Birmingham, Birmingham, AL USA; 4 Dell Medical School, University of Texas at Austin, Austin, TX USA; 5 Department of Biostatistics, University of Alabama at Birmingham, Birmingham, AL USA; 6 Department of Neurology, University of Tennessee, Memphis, TN USA; 7 Department of Anesthesiology and Perioperative Medicine, University of Alabama at Birmingham, 619 South 19th Street, JT 909, Birmingham, AL 35249-6180 USA

**Keywords:** Scholarship, Scholarly activities, Research, Graduate medical education, Curriculum

## Abstract

**Background:**

According to the Accreditation Council for Graduate Medical Education residents “should participate in scholarly activity.” The development of a sustainable, successful resident scholarship program is a difficult task faced by graduate medical education leadership.

**Methods:**

A medical librarian conducted a systematic literature search for English language articles published on scholarly activities initiatives in Graduate Medical Education (GME) between January 2003 and March 31 2017. Inclusion criteria included implementing a graduate medical education research curriculum or initiative designed to enhance intern, resident, or fellow scholarly activities using a control or comparison group. We defined major outcomes as increases in publications or presentations. Random effects meta-analysis was used to compare the rate of publications before and after implementation of curriculum or initiative.

**Results:**

We identified 32 relevant articles. Twenty-nine (91%) reported on resident publications, with 35% (10/29) reporting statistically significant increases. Fifteen articles (47%) reported on regional, national, or international presentations, with only 13% (2/15) reporting a statistically significant increase in productivity. Nineteen studies were eligible for inclusion in the meta-analysis; for these studies, the post-initiative publication rate was estimated to be 2.6 times the pre-intervention rate (95% CI: 1.6 to 4.3; *p* < 0.001).

**Conclusions:**

Our systematic review identified 32 articles describing curricula and initiatives used by GME programs to increase scholarly activity. The three most frequently reported initiatives were mentors (88%), curriculum (59%), and protected time (59%). Although no specific strategy was identified as paramount to improved productivity, meta-analysis revealed that the publication rate was significantly higher following the implementation of an initiative. Thus, we conclude that a culture of emphasis on resident scholarship is the most important step. We call for well-designed research studies with control or comparison groups and a power analysis focused on identifying best practices for future scholarly activities curricula and initiatives.

**Electronic supplementary material:**

The online version of this article (10.1186/s12909-018-1407-8) contains supplementary material, which is available to authorized users.

## Background

The Accreditation Council for Graduate Medical Education (ACGME) mandates that residents “should participate in scholarly activity” and that “[t]he sponsoring institution and program should allocate adequate educational resources to facilitate resident involvement in scholarly activities” [1]. Such broadly-defined requirements leave individual residencies to interpret and execute scholarly activities within their program in varying ways. However, this can lead to a wide diversity in residency curricula, programs, outcomes, and experiences between residency programs. As such, it is important to identify high-yield practices of successful programs so that they may be tailored to other residency programs.

It has been shown that increased research exposure and experience leads to increased fellowship acceptance and opportunities [2, 3]. Providing residents with the tools to succeed in their scholarly activities promotes the long-term benefit of producing well-rounded clinicians. Even residents who choose not to pursue academic careers will benefit from an improved ability to critically assess medical literature [4].

Given the stated importance of resident scholarly activities, medical educators are faced with the difficult task of implementing curricula and initiatives supporting residents through their scholarly experience. The availability and promotion of scholarship vastly differs between residency programs, even within the same subspecialty [5–7]. Differing opportunities for time allotted, faculty involvement, and relevant curricula can make for highly varied experiences and outcomes for residents. Therefore, it is important to describe the efforts of successful programs.

Our initial review of the literature, identified one systematic review published in 2003, focused on resident research curricula only [8]. Hebert and colleagues identified 41 articles and summarized instructional methods, goals, and objectives, as well as obstacles encountered in implementing resident research curricula [8]. They concluded that the lack of detailed developmental information and meaningful evaluations hinders educators interested in adopting a new research curriculum [8]. We set out to conduct a systematic review of the literature to extend these results beyond 2003 and to include all initiatives designed to increase intern, resident, or fellow scholarly activity.

## Methods

### Literature search

A medical librarian (ILV), who has participated in multiple systematic reviews, conducted a comprehensive literature search for English language articles published on research curricula and scholarly activities initiatives in Graduate Medical Education (GME) between January 1, 2003 and March 31, 2017 in PubMed (National Library of Medicine), EMBASE (Elsevier), and Scopus (Elsevier) databases. We chose relevant controlled vocabulary and keywords to locate GME articles focused on scholarly activities and research curricula (Additional file 1).

From the searches, 2980 unique articles were obtained. Two anesthesiology-trained authors (WW and PK) independently reviewed all titles and abstracts when available. The percent agreement on initial independent selection of articles for further review was 98.9%. Inter-rater reliability using Cohen’s Kappa was κ = 0.897, *p* < 0.001.

**Fig. 1:**
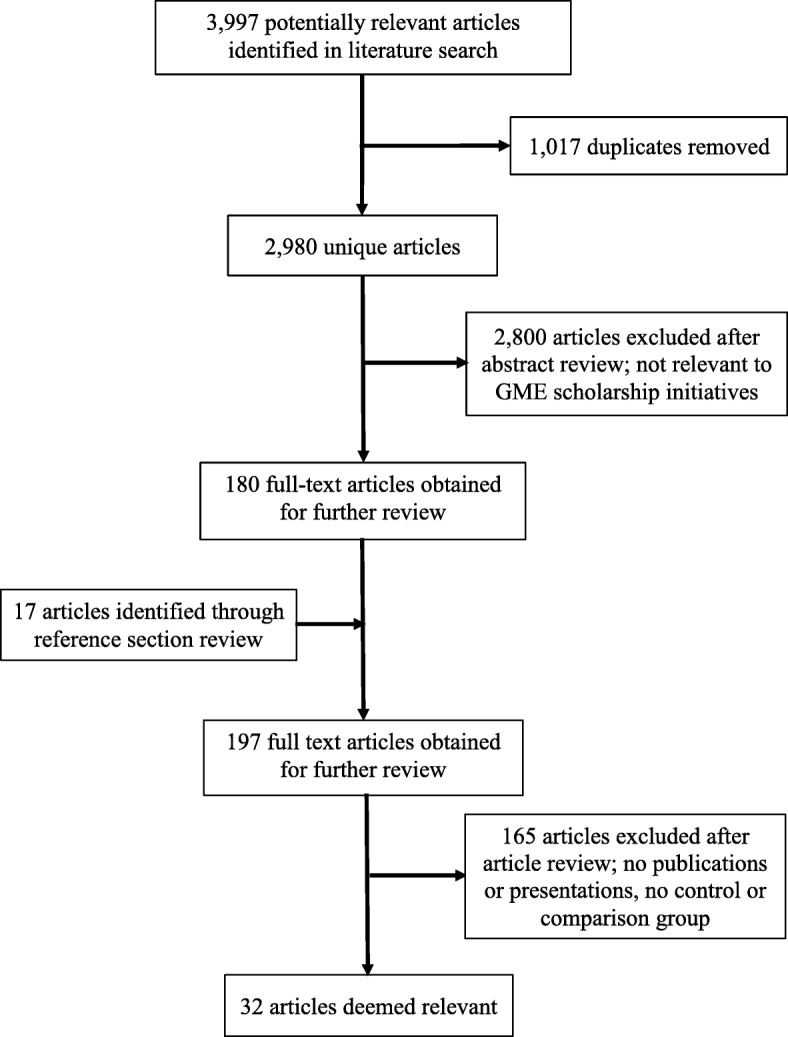
Systematic Review of the Literature on Scholarship Initiatives in Graduate Medical Education (January 2003–March 2017) Study Selection Process

When either reviewer selected an article, the full text was ordered for further review by trained research assistants (JM and AM). Research assistants also checked the reference sections of all included articles to identify other relevant studies. Using this strategy, 197 articles were obtained and reviewed for possible inclusion. All (197) articles were independently reviewed by the trained research assistants (JM and AM) to determine eligibility for inclusion. In rare cases where a disagreement occurred, the full text was reviewed by a third team member (LAR). This was followed by a team discussion of the article, where a final inclusion decision was made. After this review process, 32 articles were identified that met our protocol criteria (Fig. 1).

### Inclusion and exclusion criteria

We developed a comprehensive systematic review protocol containing operational definitions and inclusion/exclusion criteria. The operational definitions included: 1) Scholarly Activities Curriculum: defined as instruction, teaching, didactic, seminars, or workshops developed and implemented with the goal of increasing scholarly or research outcomes/productivity; 2) Initiative: defined as any activity, tactic, or intervention (e.g., role models, mentors, protected time, journal club, or project funding) implemented to improve or increase scholarly or research outcomes/productivity; 3) Major scholarly activities outcomes/productivity: defined as regional, national, or international presentation(s) and/or publication(s); 4) Participants: defined as interns, residents, or fellows in graduate medical education programs; 5) Strategies: defined as procedures or processes that the author describes as being imperative, key, or a major contributor to their study’s success; 6) Barriers: defined as obstacles or problems the author(s) described as being a hindrance or impediment to their study’s success.

Articles meeting the following criteria were eligible for review: published between January 1, 2003 and March 31, 2017; English language; studied interns, residents, or fellows of any graduate medical education discipline; implemented a curriculum or activity designed to increase scholarly activities outcomes/productivity; and presented results using a control or comparison group. Exclusion criteria included: letters to the editor, commentaries, editorials, or newsletter articles; articles that did not include a description of implementation with outcomes data; or did not measure presentations and/or publications.

### Abstraction process

Two trained reviewers (JM and AM) individually evaluated all selected articles to ascertain the study’s purpose, quality, and results. Information pertinent to the systematic review was independently abstracted, organized, and added to a spreadsheet for further assessment. Monthly meetings were held with a separate author (LAR) to review, revise, and validate the extracted data. During the course of the meetings a finalized document of abstractions was created. All abstraction disagreements were minor and were resolved during discussion between the reviewers.

### Quality assessment

We used the Medical Education Research Study Quality Instrument (MERSQI) developed by Reed et al. [9] to assess article quality. It is an 18-point, 6-domain instrument designed specifically for medical education research. The 6 domains are study design, sampling, type of data, validity of assessment instruments’ scores, data analysis, and outcomes evaluated. Since its introduction in 2007, multiple studies have shown evidence of its validity and reliability [9–11]. As described in its original use [9], the total MERSQI score was calculated as the percentage of total achievable points. This percentage was then adjusted to a standard denominator of 18 to allow for comparison of MERSQI scores across studies. One item on the MERSQI rates “type of data.” The scoring choices are “subjective, assessment by study participant” = 1 and “objective measurement” = 3. If a study measured both subjective and objective data, it was given 3 points for objective data.

### Types of data reported

The authors abstracted the following data from the selected articles: first author’s last name, year published, study location; study design, sample, and participants; program/interventions; curricula (yes/no); protected time for scholarship (yes/no); mentors (yes/no); support (yes/no); funding (yes/no); journal club (yes/no); grant writing assistance/guidance (yes/no); mandatory requirement, such as participation, attendance, completion of project, involvement in activities (yes/no); major outcomes; and price/cost. The authors defined major outcomes as publications authored or co-authored by participants or presentations at regional, national or international conferences made by participants. In addition, the authors compiled a list of strategies and barriers mentioned in the abstract or discussion sections of the included studies.

### Qualitative analysis of barriers and strategies

Barriers and strategies mentioned in either the abstract or discussion sections of the included articles were abstracted and listed in phrase format. Reviewers (JM and AM) independently created their respective lists. Reviewers then met to discuss and come to consensus on final, comprehensive lists: one for barriers and a second for strategies.

Reviewers (JM and AM) then used an immersive iterative process of content analysis [12] to identify themes and create relevant category labels. Another author (LAR) and JM then used a second iterative process to finalize category and subcategory labels.

### Statistical analysis

Descriptive statistics were used to report counts and percentages of initiatives. For each program, a publication rate, defined as the number of publications per participant per year, was estimated for the pre- and post-initiative periods. A publication rate ratio (PRR) was then calculated by dividing the post-initiative publication rate by the pre-initiative rate. A PRR greater than 1 indicates that the publication rate increased in the post-initiative period. Random effects meta-analysis [13] was then used to obtain a pooled estimate of the PRR. Meta-regression was also used to assess whether any specific initiatives were significantly associated with the publication rate.

Possible publication bias was assessed using the Egger test and with funnel plots [14]. A two-tailed *p*-value < 0.05 was considered statistically significant. All statistical analyses were performed by using R statistical software, version 3.0 [15, 16].

## Results

Our systematic review of the literature on GME scholarly activities initiatives identified 32 articles published between 2006 and 2017 (Table 1) [3, 7, 17–46]. Subjects for these articles were collected from 1989 to 2017, with 23/32 (72%) including subjects from 2000 forward [17, 18, 21–26, 28–30, 32, 34–37, 39–43, 45, 46]. Of the 32 articles in this review, 28 (88%) were United States-based [3, 7, 17–21, 23–25, 27–31, 33–39, 41–46] and 2 (6%) were Canada-based [22, 40]. The remaining locations, Germany [32] and India [26] had 1 (3%) article each. The disciplines studied were internal medicine (7, 22%), [18, 25, 27, 28, 32, 41, 42] orthopedic surgery (6, 19%), [29, 30, 33, 34, 38, 46] general surgery (5, 16%) [21–23, 31, 36], pediatrics (3, 9%) [3, 7, 40], family medicine (3, 9%) [17, 24, 45], neurology (2, 6%) [26, 39] and obstetrics and gynecology (2, 6%), [19, 37] with otolaryngology [20], gastroenterology [35], anesthesiology [43], and pulmonary critical care [44] each in one article. Nineteen (19/32, 59%) studies reported sample sizes (number of participants) [3, 7, 17, 21, 22, 25, 27, 28, 30, 32–36, 38–40, 43, 46] ranging from 25 [17] to 527 [7], with 10/19 (53%) having sample sizes less than 100 [17, 21, 25, 27, 32, 34, 35, 38, 40, 46]. Article quality scores ranged from 9.6–13.2, with a mean of 11.

**Table 1:** Brief summary of articles included in a systematic review of scholarship initiatives in graduate medical education, 2003-March 2017

Author, Year, Location	Study Design and Sample	Program/Interventions	Research Protected Time	Mentor	Major Outcomes	Price/Costs	MERSQI
Anandarajah, et al., 2016 [17] Rhode Island, USA	Pre-post study using historical data as a comparison group PGY3 Family medicine residents: 13 in comparison group (2008) vs. 12 in Primary Care Scholarly Development Program projects (2009)	1–year longitudinal PGY3 curriculum included three workshops regarding the planning of a project, six interactive seminars regarding research principles, one-to-one mentoring, and residents’ presentations at professional meetings.	No	Yes (faculty)	Increase in scholarship after implementation: • Presentation at local/regional/ national conference or publication in a peer-reviewed journal increased from 0 in 2008 to 12 in 2009	N/A	10.2
Basu Ray et al., 2012 [18] Louisiana, USA	Pre-post study using historical data as a comparison group Internal medicine residents: Comparison group (2010–2011) vs. the Consolidated Academic and Research Exposition (CARE) (2011–2012)	The Consolidated Academic and Research Exposition (CARE) program included 4 core components: house staff mentoring and the Resident Career Development Program, journal clubs, medical writing instructions, and research engagements. In addition they implemented monthly research forums and mentor meetings to discuss research related issues. Particular emphasis was given to projects that could be completed within a 1-month period and result in publication.	Yes (Research elective: 1 month in PGY1 and PGY3 or up to 2 months in PGY2)	Yes (faculty)	Increase in scholarly activity from 2010 to 2011 to 2011–2012 by 110%. • 6 submissions with 2 abstracts accepted for presentation at the Louisiana Chapter of the American College of Physicians Associates meeting (2010–2011) compared to 14 submissions, with 4 accepted for presentation (2011–2012) • 4 submissions to the Southern Hospitalist Conference (2010–2011), which increased to 7 (2011–2012), with one receiving second place in the competition	N/A	10
Brackmann, et al., 2016 [19] Michigan, USA	Pre-post study using historical data as a comparison group Comparison group (27 months before) vs. (27 months after) workgroup discussions	Biweekly, voluntary (1–2 h) gynecologic oncology research workgroup. An informal, discussion-style format for brainstorming research ideas, formulating study protocols, and collaborating on institutional review board submissions. Additional aims included editorial feedback on abstracts and manuscripts as well as oral presentation preparation. Discrete goals are set for each project by those involved such as completing a literature search, compiling a table, or writing an abstract between meetings.	2 months of protected research time	Faculty and senior residents mentor during sessions	Increase in IRB submissions, abstracts, and manuscript submissions. • IRB submissions increased from 2 to 3 after intervention • Accepted abstracts increased from 0 to 9 • Manuscript submissions increased from 1 to 6 (*p* = 0.05)	A casual dinner is provided through faculty funds	11.3
Chang et al., 2013 [20] Missouri, USA	Pre-post study using historical data as a comparison group Otolaryngology residents: Comparison group (July 1997–June 2004) vs. Reward point system (July 2004–2011)	A monetary reward point system: rewarded residents for each progressive step along the research path from project design to acceptance for publication. Each point was worth $1, which was used for allowable educational expenses (maximum of 2500 points per year).	Yes (3 month dedicated research rotation: 2-months in PGY3 and 1 month in PGY4)	No	• Mean publication output per resident per year increased from 0.13 (95% CI, 0.03–0.23) before reward system to 0.43 (95% CI, 0.26–0.60) after (*p* = 0.004) • Approved IRB projects increased from 0.47 (95% CI, 0.18–0.75) per resident per year (before reward system) to 1.29 (95% CI, 0.96–1.63) per resident per year (*p* = 0.007)	$2500/resident maximum cap $500 dollars during PGY1, and $200 during PGY2 to assist start-up	11.3
Elliott, et al., 2009 [21] California, USA	Pre-post study using historical data for intervention and comparison group General Surgery residents: 42 residents in the mandatory research group (1990–1996) and 41 residents in the voluntary research group (1999–2005)	This program switched from a required to voluntary research requirement. They evaluated if there was a significant research output difference between the two cohorts.	Yes (Elective 1–2 years of research program, Most residents choose one year option.)	No	• Publications per resident per year were statically equivalent, (Early 2.0 +/− 0.4 versus late 2.6 +/− 0.5) • Late group residents performed a higher percentage of basic science research 27.7% vs 24.4%. (P=NS) • 33% of early cohort residents continued to fellowship vs 65% of late cohort residents	N/A	11.3
Farrokhyar et al., 2014 [22] Ontario, Canada	Pre-post study using historical data as a comparison group Residents in 12 surgical specialties: 126 Research Seminar series (RSS) comparison group (2004-June 2009) and 81 Surgical Research Methodology (SRM), intervention (July 2009–2013)	SRM Program: a 2-year educational research curriculum with an orientation session and 12 modules. Education modules included readings, slides, assignments, and quizzes. PGY1 residents attended an interactive and progressive course on the principles of clinical epidemiology and basics of biostatistics presented over one year. PGY2 residents presented either a proposal or a completed study. Each resident received a program grade, based on quizzes, presentation of research, and class participation.	Yes	Yes (research methodologist and attending surgeons)	Increase in productivity and performance of the SRM residents compared with RSS residents. • SRM residents had more publications than the RSS residents (mean difference 1.0 (95% CI: 0.5–1.5; *p* < 0.001) with an effect size of 0.26 • SRM residents had more statistically significant presentations of higher evidence than the RSS residents (52.5% vs. 29%; *p* < 0.005) • Research performance improved 11.0 points (95% CI: 8.5–13.5%; *p* < 0.001) with an effect size of 0.51 in favor of the SRM residents	N/A	11.3
Fisher et al., 2010 [23] South Carolina, USA	Pre-post study using historical data as a comparison group General surgery residents	Small, inexpensive program spanning 2 years. They informed residents about faculty research, opportunities for involvement, and compiled a list of project milestones while setting semi-annual deadlines. Lectures were incorporated into the Basic Science curriculum schedule, covering the institutional review board process, case design, and statistical analysis.	No	Yes (faculty)	After the intervention there were increases in compliance, publications, and presentations. • Pre-intervention, 2 of 16 residents were compliant; post-intervention there was 100% compliance with all aspects of the program • Case report rates fell from 65, to 30%, to 25% by the end of year 2 • Increase in publications from 3 (pre-intervention) to 13 by year 2 • Increase in presentations from 5 (pre-intervention) to 13 by end of year 2	They indicated that their initiative was inexpensive, but did not provide specific amounts spent.	10
Hoedebecke et al., 2014 [24] North Carolina, USA	Pre-post study using historical data as a comparison group Family medicine residents: Research curriculum comparison group(2011–2012) vs. a resident led Scholarly Activity (SA) process (2012–2013)	Implemented a resident led SA intervention with Resident Research Teams (RRT) consisting of a volunteer PGY2 and a PGY3 with the greatest research experience among their peers. They included regular public reminders of submission opportunities, journal clubs, monthly meetings to discuss goals as well as to share ideas, pairing of interns/students with senior mentors with similar interest, and publically recognize scholarly accomplishments.	Yes (4 h per month, 4 week PGY-1 research rotation)	Yes (senior residents and faculty)	After the resident driven process there were increases in presentations, publications, and involvement. • Increase in the number of presentations from 3 (2011–2012) to 28 (2012–2013): 7 regional, 10 national, and 11 international presentations • Increased resident peer-reviewed publications from 2 (2011–2012) to 6 (2012–2013)	N/A	10
Kanna, et al., 2006 [25] New York, USA	Cohort study using concurrent data as comparison groups Internal medicine residents: 38 non-rotators comparison group and 43 research program rotators intervention (May 2004)	A two-week structured research rotation included dedicated faculty and online resources. Intensive 2-h weekly sessions on research methodology were conducted for four weeks every year. Residents were provided dedicated time to attend didactic lectures. All residents were required to participate and complete at least one scholarly activity during their training period. The research director was given protected time to administer, teach, and evaluate all research components. The research director conducted research-in-progress seminars and had daily meetings with the residents during their research rotation.	Yes (2 week research rotation with no clinical obligation)	Yes (A qualified faculty member with a Masters of Public Health was appointed as research director)	There was an increase in total research projects, letters to the editor, and publications among rotators vs. non-rotators. • The rotators group outperformed the non-rotators group in total research projects (published & non-published) (*p* < 0.001) and letters to editors (*p* < 0.001) • The number of residents who were able to publish among the rotators was marginally significant compared with non-rotators (*p* = 0.053) • Significantly more participation by rotator residents in scholarly activities (*p* < 0.001)	N/A	12
Khurana et al., 2015 [26] Chandigarh, India	Pre-post study using historical data as a comparison group Neurology residents: Pre-publication comparison group (June 2010–April 2012) vs. publication rotation intervention (June 2012–September 2013)	A publication rotation was created with mandatory participation of residents. There was no formal class on research development. Residents were encouraged to analyze landmark research trials, review articles, and send Letters to the Editor (correspondence) if they found flaws or had a differing opinion.	Yes (15 day rotation per 6 months)	Yes (faculty)	The number of publications by residents increased after the research publication rotation, with a significantly greater amount in high impact journals. • Total resident publications increased from 10 (pre-rotation period) to 27 (post-rotation period) • 1 paper was accepted by a high-impact journal (pre-rotation period) compared to 9 papers (post-rotation period) • The total impact factor increased from 22.68 to 116.66 (*p* = 0.039) • 11 clinical images were published (post-publication rotation) compared to 1 (pre-publication rotation) • The number of original articles produced was the same in both periods	N/A	11.3
Kohlwes, et al., 2006 [27] California, USA	Cohort study using some historical and concurrent data as comparison groups 32 internal medicine residents from 2000 to 2004 vs. historical comparison group (1990’s), as well as residents not in the Primary Medical Education (PRIME) program	The PRIME program was a two-year outpatient based internal medicine residency track. The program accepted 8 residents per year who divided their time evenly between the standard categorical inpatient rotations and the PRIME outpatient blocks. The PRIME curriculum consisted of didactic lecture, weekly journal club, work-in-progress sessions, and active mentoring. Didactic time was divided evenly between clinical outpatient topics, behavioral medicine, and epidemiology/ research methods training. Faculty lectured, facilitated work-in-progress sessions, and had monthly meetings with residents.	Yes (2 half days of didactic time, and 1–2 half days of research time)	Yes (faculty)	Increases in resident competency and local presentations were seen after implementation of PRIME. • The overall clinical competence scores of the 32 PRIME residents were 8.23 on a 9.0-point scale vs. 8.09 for the rest of the internal medicine program (*p* < 0.001) • 22 of 32 PRIME residents (2001–2004) produced 20 original research papers published or in-press in peer-reviewed journals, 2 clinical-review papers, 1 paper currently under review, and 2 book chapters • Resident presentations at the Rector Symposium rose from 6 to 9 per year in the 1990’s to 13–21 (2001–2004)	N/A	11.3
Kohlwes, et al., 2016 [28] California, USA	Cross-sectional survey 71 PRIME and 98 non-PRIME (comparison) internal medicine residents from 2001 to 2010	This track immerses residents in a clinical outcomes research curriculum and provides opportunities for participants to conduct research projects. The curriculum addresses several barriers to research productivity during residency. We ensure baseline knowledge of research methods through resident participation in a Designing Clinical Research class offered in person or online. Ongoing didactic and work-in-progress sessions continue year-round on ambulatory blocks to support resident research and help ensure success.	Protected time for research occurs during the 6 months of ambulatory medicine training time scheduled for every resident during the 2nd and 3rd years of residency.	Yes (faculty)	An individualized learning pathway enables residents to successfully publish manuscripts and access mentorship. • PRIME alumni were more likely to publish their residency research projects compared to non-PRIME alumni (64% vs. 40%, *p* = 0.002) • Strongly agree to adequate access to mentors during residency for PRIME vs. non-PRIME (4.4/5 vs. 3.6/5, *p* = 0.001) • 63% of PRIME alumni agreed that their research experience during residency influenced their subsequent career choice versus 46% of non-PRIME alumni (*p* = 0.023)	N/A	10.2
Konstantakos et al., 2010 [29] Ohio, USA	Pre-post study using historical data as a comparison group Orthopedic residents: Pre-intervention comparison group (September 2000–August 2005) vs. a Research Team (RT) (September 2005–November 2009)	Creation of a RT that encompassed a research director, faculty, research residents, biostatistician, and other support staff that oversaw residents’ scholarly activity during monthly meetings. Mentors met weekly with, collaborated, guided, and evaluated the resident. Throughout the academic year, all residents were provided with dedicated time to attend a lecture series on planning and conducting research, biostatistics, and critical appraisal of the medical literature taught by public health and statistics faculty. Attention was also focused on seeking external funding. A manuscript that was ready for, or submitted to, a peer-reviewed journal was a requirement for graduation.	Yes (3 months of PGY2 with 2 half days of clinical duty per week and 2–3 days of night call per month)	Yes (faculty)	Scholarly activity increased substantially from the 5-year period before the implementation of the RT to the 4-year period after initiation of the RT. • Peer-reviewed publications on which residents were authors increased from 1 to 10 per year • Peer-reviewed national presentations at professional meetings increased from 6 to 11 per year • Peer-reviewed local and regional presentations increased from 2 to 4 per year	Departmental Reimbursements based on FTE: Biomechanical engineer 30%; Statistician 10%; Administrator 10%; Basic scientist 10%; Research coordinator 100%	10
Krueger, et al., 2017 [30] 3 residencies from unknown locations in the USA	3 nonrandomized groups: 3 orthopedic residencies with varying amounts of protected research time in each residency, retrospectively evaluated between 2007 and 2014	Peer-reviewed publications from 3 residency programs were examined retrospectively from January 2007 through December 2014. All 3 programs shared the same research requirement—completing at least 1 publishable research project during residency.	Program 1: research year is mandatory for all residents and is completed between their PGY3–4 years. Program 2: all residents have the opportunity to volunteer for an elective research year between their PGY2–3 years. Program 3: no residents were given protected research time during their residency	No	• Residency programs with dedicated research time did not produce significantly (*p* > 0.05) more, or higher quality, peer-reviewed publications than residencies without dedicated research time. • There was no significant difference in the number of publications produced between the institutions on a per-staff (*p* = 0.27) or per-resident (*p* = 0.80) basis. • There were no significant differences between programs in terms of the SCImago Journal Ranking for the journals containing their publications (*p* = 0.135).	N/A	12.7
Kurahara et al., 2012 [3] Hawaii, USA	Pre-post study using historical data as a comparison group Pediatric residents: 63 pre-intervention comparison group (1994–2000) and 72 Residency Research Requirement and Program (RRRP) residents (2001–2007)	RRRP focused on increased resident productivity, faculty collaboration, and seeking fellowship training. Elements included didactic lectures on research and journal clubs for critical review of the literature. There was a research week for resident presentation. Most residents conducted research during PGY2 or PGY3 when more time was available. Faculty aided by re-writing articles and in fielding arguments in the peer-reviewed journals.	No	Yes (faculty)	There was a significant increase in publications, collaboration, and scholarly activity. • Publications increased from 2 to 26 post intervention (*p* < 0.001) • Collaboration between resident co-authors increased from 5 to 31 post intervention (*p* < 0.001) • 8% of residents involved with published research pre-intervention to 36% during RRRP • Pediatric faculty involvement in resident projects increased significantly from 1 to 48 (*p* < 0.001) • Pre-intervention 18% of residents who went into fellowship published vs. 4% of those who went into general pediatrics; increased post-intervention to 63% of those who went into fellowship vs. 23% of those in general pediatrics	N/A	11.3
Lohr, et al., 2006 [31] Ohio, USA	Pre-post study using historical data as a comparison group General surgery residents: Pre-intervention comparison group (1989–1997) vs. a Research Team (RT) (1997–2003)	Each RT consisted of a faculty mentor, senior resident, mid-level resident, and an intern. Residents maintained their team assignment throughout their 5-year appointment. An annual 6–8 week structured lecture series, including critical thinking, evaluation of the literature, statistics, guidelines for research projects, literature searches, hypothesis development, and tools for data collection. Journal clubs were held to improve resident literature reading skills, analytical review, research methods, biostatistics, and epidemiology. Support staffs such as a research director, research nurses, epidemiologist, and research specialists were available to assist the RT. Residents were expected to submit a case report and present at a national or regional meeting.	No	Yes (faculty and senior residents)	The RT increased the amount of presentations, publications, and peer-reviewed publications though none met statistical significance. • Pre-RT, 69 presentations and 60 publications compared to 92 presentations and 77 publications after the RT • Peer-reviewed publications were 83% pre-RT compared with 92.5% of publications post-RT • The RT resulted in a 33% increase in presentations and a 13% increase in publications	The Institute received an endowment from Dr. E. Kenneth Hatton in 1997. However, the amount used for resident research efforts was not specified.	10
Löwe, et al., 2007 [32] Heidelberg, Munich, and Tubingen, Germany	Cohort study using concurrent data as comparison groups Internal medicine, psychotherapy and psychosomatics, psychiatry, and psychology residents: 22 non-participant comparison group and 15 Resident Training Program (RTP) participants (2005)	Structured 1-year training program with three elements: 1) provision of a methodological research knowledge within the scope of a “Clinical Research Methods” course; 2) mentorship by an experienced researcher; and 3) an individual research project. The Clinical Research Methods course had 33 weekly lectures, each lasted 90 min.	No	Yes (faculty)	Residents within the training program had increased scholarly activity, presentations, and grant applications. • Significantly more intervention subjects were currently working on a journal article (87% vs. 36%; *p* = 0.003) • RTP residents had increased presentations of scientific results at research meetings (80% vs. 41%; *p* = 0.04) • Increased RTP completion of 1 original paper as coauthor (60% vs. 18%; *p* = 0.01) • RTP residents completed significantly more applications for funding as co-investigators (47% vs. 5%; *p* = 0.004) • Significantly more RTP grant applications were accepted for funding (33% vs. 0%; *p* = 0.007)	Annual research money per full professor (438,000€, 458,000€, and 484,000€,)	13.2
Macknin, et al., 2014 [33] Ohio, USA	Cohort study using concurrent data and retrospective data: 48 research track and 74 traditional track residents Orthopedic residents: Compared the outcomes of 74 traditional track and 48 research track training from 1987 to 2006.	Created two research tracks for orthopedic residents. Research track residents were given 1 year for full-time basic science research after their intern year. Traditional residents completed a research project during residency with significantly less protected time and no long term protected time.	Traditional track—no Research track: 1 year of dedicated research time	Yes (orthopedic scientist)	Residents in the research track were more likely to publish during residency and throughout their careers. • Residency publications were higher for research track than traditional residents (71% vs. 41%, *p* < 0.01) • Mean publications during residency for research residents were higher than traditional (1.8 vs. 0.9 publications) • Publications after residency were higher for research residents than traditional (7.7 vs. 4.8 publications.) • Residents who published in residency were more likely to continue publishing in their careers compared to those who did not (75% vs. 55%, *p* = 0.02)	N/A	12
Manring, et al., 2014 [34] Ohio, USA	Pre-post study using historical controls as a comparison group Orthopedic residents: 42 pre-intervention (2006–2008) and 46 orthopedic residents with a multifaceted publications program (2009–2012)	Appointed a research and curriculum director to increase output of research by requiring: 1) preparation of a review article or systematic review; 2) presentation of a clinical or laboratory research project; 3) preparation and submission of a manuscript based on a research project to a peer-reviewed journal; and 4) mentoring of a junior resident on research. A research editor assisted with publication and manuscript production. Faculty members mentored and delivered lectures on research methodology, statistical analysis, and regulations. Regular meetings were established to monitor topic developments, presentations, findings, and submissions to granting agencies that focus on orthopedic resident research.	No	Yes (faculty and senior residents)	There was a rise in authorship, publications, and presentations among residents. • Resident publications in peer-reviewed journals increased from 6 (2009) to 53 (2012) • Resident presentations at national or international conferences increased from 15 (2008–2009) to 24 (2009–2010), and were 19 (2011–2012)	Metropolitan area technical editor described as $50,000 to $60,000 per year.	11.3
Mayo, et al., 2015 [35] Texas, USA	Pre-post study using historical data as a comparison group Gastroenterology fellows: 29 Pre-program comparison group (2001–2007) and 43 research program intervention (2008–2014)	Creation of a structured research program that emphasized clear expectations, protected time, mentorship and oversight, support via a statistician, an educational curriculum, tracking of accomplishments, and accountability. They instituted a fellowship research committee that oversaw and ensured residents were completing research in a timely manner. Fellows met with the committee semi-annually. Fellows met with faculty mentors bimonthly. The educational curriculum consisted of online modules and 6 specific lectures: 1) How to select a mentor; 2) How to select a project; 3) How to get your article published; 4) Statistics for the trainee; 5) Database searches and electronic journals; and 6) Use of reference management software	Yes (1 month in year 1, 3 months in year 2, and 2 months in year 3)	Yes (faculty)	There was an increase in publication, scholarly activity, and pursuit of an academic career. • Trainees presenting original research at scientific meetings or publishing their work in peer-reviewed journals increased from 33 to 100% • Fellows meeting the scholarship requirement increased from 20 to 60% and by the 3rd year, the research metric was achieved in 100% of fellows • The number pursuing careers in academic medicine increased approximately 3-fold, from 14 to 51%	Authors funded 10% of the statistician’s effort or if they were funded they could pay an hourly rate.	9.6
Mills, et al., 2011 [7] North Carolina, USA	Retrospective cohort study using concurrent data as comparison groups Pediatric residents and fellows: 295 non-exposed comparison group and 232 exposed to an Evening of Scholarship (EOS) (1985–2007)	A pediatric program created a voluntary EOS that allowed residents and fellows to present their research on an annual basis to the department. They sought to measure the future publication rate of residents who engaged with EOS against those who did not participate (1985–2007).	No	No	Residents and fellows involved with EOS were more likely to publish in the future than those that were not engaged. • 69% of EOS residents had publications after graduation versus 34% of nonparticipants (*p* < 0.001) • Participants in EOS were more likely to have previously published (31% vs. 15%; *p* < 0.001) • EOS residents had more publications after training (*p* < 0.001)	This project was supported through grant funds. However, amounts used toward resident research efforts were not specified.	11.3
Papasavas, et al., 2013 [36] Connecticut, USA	Pre-post study using historical data as a comparison group General surgery residents: 60 pre-intervention comparison group (July 2008 to June 2010) and 58 research requirement residents (July 2010–June 2012)	Research program consisted of a research curriculum, an annual research day, research mentors, project repository, statistical support, a Director of Research, and data base mining. Monthly meetings and lectures incorporated into the overall resident core curriculum. They invited IRB staff; statisticians; and senior scientists involved in basic science, translational, and outcomes research to lecture residents. During these meetings, there were opportunities for the residents to discuss the design of their research project and get feedback from the faculty and fellow residents There was a requirement to submit an abstract 30 days before a meeting with specification similar to national meetings regarding abstract structure and word limit. There was also an alternate 2-year research fellowship available at the end of PGY2.	No	Yes (faculty)	With the creation of a research requirement there was an increase in poster and podium presentations. • Increase from 9 of 60 (15%) residents with a podium or poster presentation to 23 of 58 (40%) (*p* < 0.01) • Significant increase in the proportion of podium presentations at national/international vs. regional meetings (*p* < 0.01) • Increase from 14 residents producing 31 publications in peer-reviewed journals to 17 residents producing 32 publications • 88% of 58 podium and poster presentations post-intervention originated from residents who participated in the research requirement • Residents in the 2-year research track produced significantly higher proportion of publications (62% vs. 19%; *p* < 0.05)	Various departments of surgery covered the expense of statistical support. However, amounts used toward resident research efforts were not specified.	11.3
Penrose, et al., 2012 [37] Texas, USA	Pre-post study using historical data as a comparison group Obstetrics and gynecology residents (12 total residents in residency): Pre-intervention comparison group (2007–2008) vs. Baby Steps Program (BPS) (2008–2010)	An obstetrics and gynecology program added dedicated research staff to facilitate and coordinate resident research projects, and support clinical faculty in research activities. Faculty concentrated their efforts on developing research ideas and mentoring resident researchers with the assistance of the post-doctoral researcher to coordinate research efforts.	No	Yes (faculty and research coordinator)	• Resident presentations rose from 2 regional/national to 8 regional and 4 national presentations • 8 of 12 clinical faculty members were engaged as mentors in resident research compared with only 3 in past years	All faculty salaries are funded through clinical efforts, not research dollars. The postdoctoral researcher position was created and filled in August 2009 using clinically generated Funds. However, amounts used toward resident research efforts were not specified.	10
Robbins, et al., 2013 [38] New York, USA	Pre-post study using historical data as a comparison group Orthopedic residents: 75 pre-curriculum comparison group (1998–2006) and 32 research curriculum intervention (2007–2010)	Structured program included research milestones for each training year, a built-in support structure, use of an accredited bio-skills laboratory, mentoring by National Institutes of Health–funded scientists, and protected time to engage in required research or prepare scholarly peer-reviewed publications. 8 h of lecture per year. Topics included research design, navigating the IRB process, critical appraisal, and basic research methods (i.e., statistical design). Residents had the option to pursue a research year free from clinical training to gain additional academic and/or research experience.	Yes (6–7 weeks or 960 h per year)	Yes (faculty and research coordinator)	• The total amount of grants awarded increased from $15,000 for eight 2007 graduates to $380,000 for nine 2010 graduates • Residents began to submit more than one research proposal, peaking in 2010 with 9 graduates submitting 17 applications • The twelve 2005 graduates had a total of 16 publications from 2000 through 2006, whereas the nine 2010 graduates published 84 papers from 2005 through 2011 • 162 publications from the 1998–2006 graduates (9 years) and 341 publications from the 2007–2010 graduates (4 years)	The costs per year included $19,000 (0.3 FTE) for an academic research coordinator; $16,000 for resident travel to professional meetings; reimbursement for 213 faculty hours; and funding for resident salaries while on the research rotation, paid by the hospital budget.	10
Robbins, 2017 [39] New York, USA	Pre-post study using historical data as a comparison group Neurology residents: 53 pre-program (2005–2009) vs. 57 post-program (2011–2015)	Components included an expanded journal club led by 2 investigators during which resident projects were discussed in workshop form, guided mentorship provided, a required grand rounds platform presentation before graduation, and the presentation of annual awards for the most scholarly and seminal research findings, as judged by a faculty awards committee. Required the production of peer-reviewed publications, presentations at scientific meetings, and authored book chapters or textbooks. The program was formally administered by an associate residency program director.	Could use electives to complete research project	Yes (faculty)	Research outcomes increased after the program was implemented. • Percentage of first-authored abstract presentation or publication of residents increased from 30.2 to 71.9% (*p* < 0.0001) • Abstracts per resident increased from 0.15 +/− 0.41 to 1.26 +/− 1.41 (*p* < 0.001) after intervention • Peer-reviewed publications did not show a statistically significant increase pre- to post-program (*p* = 0.36)	N/A	11.3
Roth, et al., 2006 [40] Alberta, Canada	Pre-post study using historical data as a comparison group Pediatrics residents: 20 pre-curriculum comparison group (2002–2003) and 23 research curriculum intervention (2004–2005)	Research curriculum had three main components: 1) the resident research project; 2) a supportive training environment; and 3) accessible research funding. Twenty, 75-min academic sessions addressed the steps of a research protocol. Residents directed monthly journal clubs. During research blocks residents did not have daytime clinical duties and had reduced nighttime call responsibilities. Mentors guided the resident through study design, conduct and analysis, and obtaining funding. Medical librarians assisted with literature searches and departmental biostatisticians provided consultation to residents. Residents were required to present their work at least once during residency.	Yes (12 weeks during PGY1 and up to 8 weeks PGY2-PGY4)	Yes (faculty)	There was an increase in all measures from the comparison group to the intervention, but none of the observed differences were statistically significant. • Research articles increased from 3 to 5 • Research grants increased from 3 to 7 • Conference presentations increased from 6 to 9	Departmental funds allocated to resident research grants include seed money ($250 CDN) allocated on a non-competitive basis to offset the administrative costs associated with preparation of a research proposal. Residents could receive $2000 CDN per project per year to present at meetings.	11.3
Rothberg, et al., 2014 [41] Massachusetts, USA	Pre-post study using historical data as a comparison group Internal medicine residents: Pre-implementation comparison group (2001–2006) vs. implementation of a Resident Research Program (2006–2012)	The program consisted of evidence-based medicine training, 4 two-hour interactive workshops, to stimulate interest in research. Structural changes were made to support resident’s conduct of research including protected time during ambulatory blocks, a research assistant who aided with tasks such as institutional review board applications and data entry, a research nurse to assist with data collection, easily accessible biostatistical support, and a resident research director to provide mentorship.	Yes (1 day per week PGY2)	Yes (faculty and research director)	• Resident publications increased from 3 to 58 (*p* < 0.001) • Residents accepted to subspecialty fellowships increased from 28 to 50 (33 to 49%; *p* = 0.04) • Original research increased from 2 to 28 • Residents first authors increased from 1 to 33	0.25 FTE for research director; 0.5–1.0 FTE for master’s-level statistician; 10 h per week for research nurse; $750/ resident who presents at a national meeting; $750 in prizes; and $175 for catering	10.8
Ruiz, et al., 2011 [42] North Carolina, USA	Pre-post study using historical data as a comparison group Internal medicine residents: Pre-intervention comparison group (July 2006–June 2007) vs. Research Curriculum intervention (July 2007–June 2009)	Developed a comprehensive 3-year curriculum, appointed a chief resident for research and a faculty research director to coordinate all resident research activities. All residents were involved in a 3-year evidence-based medicine curriculum that covered 12 to 14 topics in research methodology, statistical methods, research design, and manuscript preparation. There were monthly research forums to discuss projects and future directives. Residents were required to submit an abstract and present a poster of their work at the annual departmental research day.	Yes (1–3 months of research elective time per year; not to exceed a total of 3 months)	Yes (faculty and research chief resident)	• Graduates with a peer-reviewed publication increased (7% vs. 32%; *p* = 0.04); publications increased from 7 (comparison group) to 11 (year 1) to 15 (year 2) • Graduates with a presentation at a national meeting increased from 5 to 10 (4% vs. 29%; *p* = 0.02) • Regional presentations increased from 2 to 9	The department committed to providing funding for poster printing and meeting-related expenses, including costs of transportation, lodging, and meals for regional and national meetings when not provided by the sponsoring subspecialty section or a third party. However, amounts used toward resident research efforts were not specified.	11.3
Sakai, et al., 2014 [43] Pennsylvania, USA	Pre-post study using historical data and rank-to-match analysis as a comparison group Anesthesiology residents: 54 Pre-initiative comparison group (2003–2006) and 65 post-initiative intervention (2007–2011)	An annual research introductory lecture was given (1-h lecture of basic grantsmanship, steps in research activity, and introduction of potential faculty research mentors), deadlines for abstracts and meetings were presented, scholarly achievements were announced on the department website, the Resident Research Rotation (RRR) director was appointed, a 90-min research problem–based learning discussion was developed, and an annual Trainees Research Day was formed. The following initiatives were implemented only for the PGY4 elective RRR: a formal application process for acceptance, mandated attendance at a weekly research meeting with the rotation director where weekly milestones were presented, and submission of an abstract to local and state resident research competition was strongly recommended.	Yes (6 months for senior residents)	Yes (faculty and research director)	• Resident peer-reviewed publications went from 16 pre- to 41 post-intervention • The RRR residents (*n* = 25) published 36 articles whereas the non-RRR residents (*n* = 94) published 33 articles • The RRR residents authored more original articles than the non-RRR residents (83.3% vs 33.3%, *p* < 0.0001) • A greater number of post-initiative residents entered fellowships (42.6% vs. 70.8%; *p* = 0.002)	N/A	11.3
Schnapp, et al., 2009 [44] Washington, USA	Pre-post study using historical controls as a comparison group Pulmonary and critical care medicine (PCCM) fellows: Pre-intervention comparison group (1995–2000) vs. Translational Research Training Program (TRTP) intervention (2001–2006)	Increased collaboration between clinical and basic science researchers. Research training in the primary research discipline of a trainee (basic science or clinical), cross-training in the alternate research discipline, development of a research project that included a translational research component, and enhancement of the research environment to emphasize translational research. All fellows are required to complete a research project under the direction of a faculty mentor and the mentoring committee. There was a 9-week course that covered basic research methods, a laboratory workshop, and statistical approaches. Modified journal clubs evaluated literature from a basic science and clinical standpoint.	No	Yes (faculty)	• The average number of authors per manuscript increased from 3.79 in 1995 to 5.54 in 2006 (*p* < 0.05) • The total number of publications from PCCM Division members increased from 43 (1995) to 52 (2000) and 83 (2006) • Average number articles published per fellow prior to institution of TRTP (1995–2000) was 2.5, and after institution of TRTP (2001–2006) it increased to 4.7 • Publications that included PCCM Division members from more than one training track increased from 8% in 2000 to 32% in 2006 (*p* < 0.005) • The percent of translational articles was similar in 1995 and 2000 (9 and 11%, respectively), but increased to 22% in 2006 (*p* > 0.05)	N/A	11.4
Seehusen, et al., 2009 [45] Georgia. USA	Pre-post study using historical data as a comparison group Family medicine residents: Pre-point system comparison group (2002-June 2006) vs. Point System intervention (July 2006–July2007)	Research point system tabulated resident’s research productivity. Residents had to accumulate 10 scholarly activity points. The research director, program director, and faculty mentor determined point allotments. The most efficient way to accumulate points was through a research project and subsequent presentation. Residents were allowed to collaborate with one another or faculty on projects.	No	Yes (faculty)	• 4 peer-reviewed medical journal publications pre-intervention compared to 4 during the post-intervention period • Regional, national, and/or international podium/poster presentations increased from 5 to 8 • Increase from 1 book section or chapter to 5 with resident authors	N/A	10
Torres, et al., 2015 [46] Texas, USA	Pre-post study using historical data as a comparison group Orthopedic residents: 24 pre-implementation comparison group (2001–2006) and 27 dedicated resident research program intervention (2007–2012)	The dedicated resident research program included: 1) the requirement for the number of original research projects per resident increased from one to two; 2) each project required at least one faculty mentor; 3) a project proposal had to be reviewed by the newly established departmental research committee and revised as needed; 4) the resident presented the project proposal to the entire departmental faculty for majority approval before it was accepted as an official project; 5) once the project was approved, the research committee monitored the project’s progress; and 6) project completion was achieved by manuscript submission for peer-reviewed publication.	No	Yes (faculty)	• Post-intervention residents published more papers during residency than the comparison group [1.15 vs 0.79 publications per resident; 95% CI (0.05,0.93); *p* = 0.047] • Journal impact factor increased after implementation [1.25 versus 0.55 per resident; 95% CI (0.2,1.18); *p* = 0.005] • Trainees after implementation more often continued education in a subspecialty fellowship (81.5% versus 45.8%; *p* = 0.008)	N/A	11.3

*PGY* Postgraduate Year

*CI* Confidence Interval

*IRB* Institutional Research Board

*USA* United States of America

*N*/*A* Not applicable

*FTE* Full Time Equivalent

*CDN* Canadian Dollar

*NS* Non-significant

*MERSQI* Medical Education Research Study Quality Instrument

### Initiatives

Nineteen (19/32, 59%) articles included a curriculum focused on research topics [3, 17, 18, 22, 23, 25, 27–29, 31, 32, 34–36, 38, 40, 42–44]. Eight articles provided 4–28 lectures (60 to 120 min each) offered over 4 weeks to two years [17, 22, 25, 27, 31, 35, 42, 44]. The entire didactic experience could encompass a 2 week block [18] or continue throughout residency [3].

Sixteen (84%) of the studies with curriculum provided details about the didactic topics covered [17, 18, 22, 23, 25, 27, 29, 31, 32, 34, 35, 38, 40, 42–44]. Of these, statistics (11/16, 69%) [18, 22, 23, 25, 29, 31, 32, 34, 35, 38, 42] and research design (12/16, 75%) [17, 18, 23, 25, 27, 29, 31, 32, 34, 38, 40, 42] were the most frequently reported, followed by critical appraisal of the literature (6/16, 38%), [25, 29, 31, 32, 38, 42] Institutional Research Board (IRB) and ethics (7/16, 44%), [17, 18, 23, 27, 34, 38, 42] epidemiology (3/16, 19%), [18, 22, 27] searching the literature (3/16, 19%), [25, 35, 42] and formal writing (3/16, 19%) [32, 35, 42]. Less frequently reported topics included: research pearls [42], outcomes research [36], critical thinking [31], funding options [43], research career advancement [42], how to get your article published [35], tips for completing research projects [42], and overcoming procrastination [42].

Under half of the studies with curriculum (7/19, 37%) reported using needs assessment prior to the development of their research curriculum. Two conducted surveys [25], and one program held a faculty retreat [35] to construct their needs assessment. Another used both interviews and a committee [40]. The remaining 4 studies (21%) used a committee or team that discussed and developed the curriculum [17, 34, 38, 42]. Ten (10/19, 53%) studies evaluated the curriculum they implemented via surveys [17, 18, 22, 25, 28, 32, 33, 35, 44], quizzes/exams [22, 32] and/or interviews [17, 35, 38]. All but one of these articles reported results of the evaluation [38]. All surveys and interviews reporting participant satisfaction and/or confidence demonstrated support for the research curriculum [17, 22, 25, 28, 32, 35, 44], except one, which had mixed results [18]. The two studies testing knowledge [22, 32] compared results of a control group to an intervention group (received curriculum) showing statistically significant results in favor of the curriculum. In addition, one of these studies used a pre-post design as well as the comparison to a control group [32].

Most included articles (31/32, 97%) used multiple interventions with the goal of increasing scholarly productivity [3, 17–46]. The one article that did not use multiple interventions used an annual research day to stimulate an increase in research productivity [7]. The number of interventions ranged from 1 [7] to 8 [40] [42], (mean 4.0 ± 1.7; median 4).

The majority of studies provided residents with mentors (28/32, 88%) [3, 17–19, 22–29, 31–46]. Over half incorporated protected time (19/32; 59%) [18–22, 24–30, 33, 35, 38, 40–43] However, protected time differed between residencies and ranged from a 2 week rotation [25] to a 1 year research elective. [21, 33] Fifty-six percent included a mandatory initiative (18/32), such as required attendance [18, 22], participation [3, 21, 23, 24, 26, 30, 32], or completion of a project [17, 19, 25, 27, 36, 40, 42, 44, 45]. Journal clubs were described in 41% (13/32) of studies [3, 18, 24, 25, 27, 31, 38–44] and 31% (10/32) provided assistance or guidance on grant writing and/or application. [18, 19, 29, 31, 34, 37, 38, 40, 42, 43] Funding was available for participants in only 25% (8/32) of studies. [20, 29, 31, 33, 38, 40, 42, 46]

While almost half of the studies (15/32, 47%) provided some information relevant to cost of the program (Table 1) [7, 19, 20, 23, 29, 31, 32, 34–38, 40–42], these statements tended to be vague failing to address the critical factors of feasibility and sustainability. The most detailed description came from, Robbins et al. [38] who approximated their per-year costs to be $19,000 for an academic research coordinator, $16,000 for resident travel to professional meetings, reimbursement for 213 faculty hours and funding for resident salaries while on the research rotation. Unfortunately, even these costs are outdated, as they came from expenses incurred during academic years 2007 to 2010.

### Major outcomes

Our primary outcomes were publications and presentations. However, only 25% (8/32) of articles explicitly required participants to achieve a specific outcome such as submission of a scholarly manuscript [29, 34, 38, 39, 46] or a regional, national, or international presentation [31, 35, 41]. Despite this, the majority of articles (29/32, 91%) reported on resident publications, [3, 7, 19–36, 38–46] with 28/29 (97%) reporting on peer-reviewed publications [3, 7, 19–22, 24–36, 38–46]. Of those, 10 (36%) reported a statistically significant increase in their publication rate after implementation or changes made to a scholarship initiative [3, 7, 20, 22, 32, 33, 36, 41, 43, 46]. More than half (16/28; 57%) of the publications were reported as original research, [21, 22, 25–28, 30, 32–35, 39, 41, 43, 45, 46] 6/28 (21%) as case reports, [26, 31, 34, 39, 43, 45] and only 5/28 (18%) as book chapters [27, 32, 39, 43, 45]. Fifteen (15/32, 47%) articles reported on regional, national, or international presentations [17, 18, 22–24, 29, 31, 32, 34, 36, 37, 39, 40, 42, 45], with only 2/15 (13%) reporting a statistically significant increase in presentation rates [22, 36]. One article combined publication and presentation rates to obtain statistical significance [39]. Overall, 30 articles reported a positive increase in resident publications and/or presentations after implementation of a scholarly activity initiative [3, 7, 17–20, 22–29, 31–46]. However, 21/32 (66%) of the included articles did not report a statistically significant increase in either presentations or publications [17–19, 21, 23–31, 34, 35, 37, 38, 40, 42, 44, 45].

### Meta-analysis

**Fig. 2:**
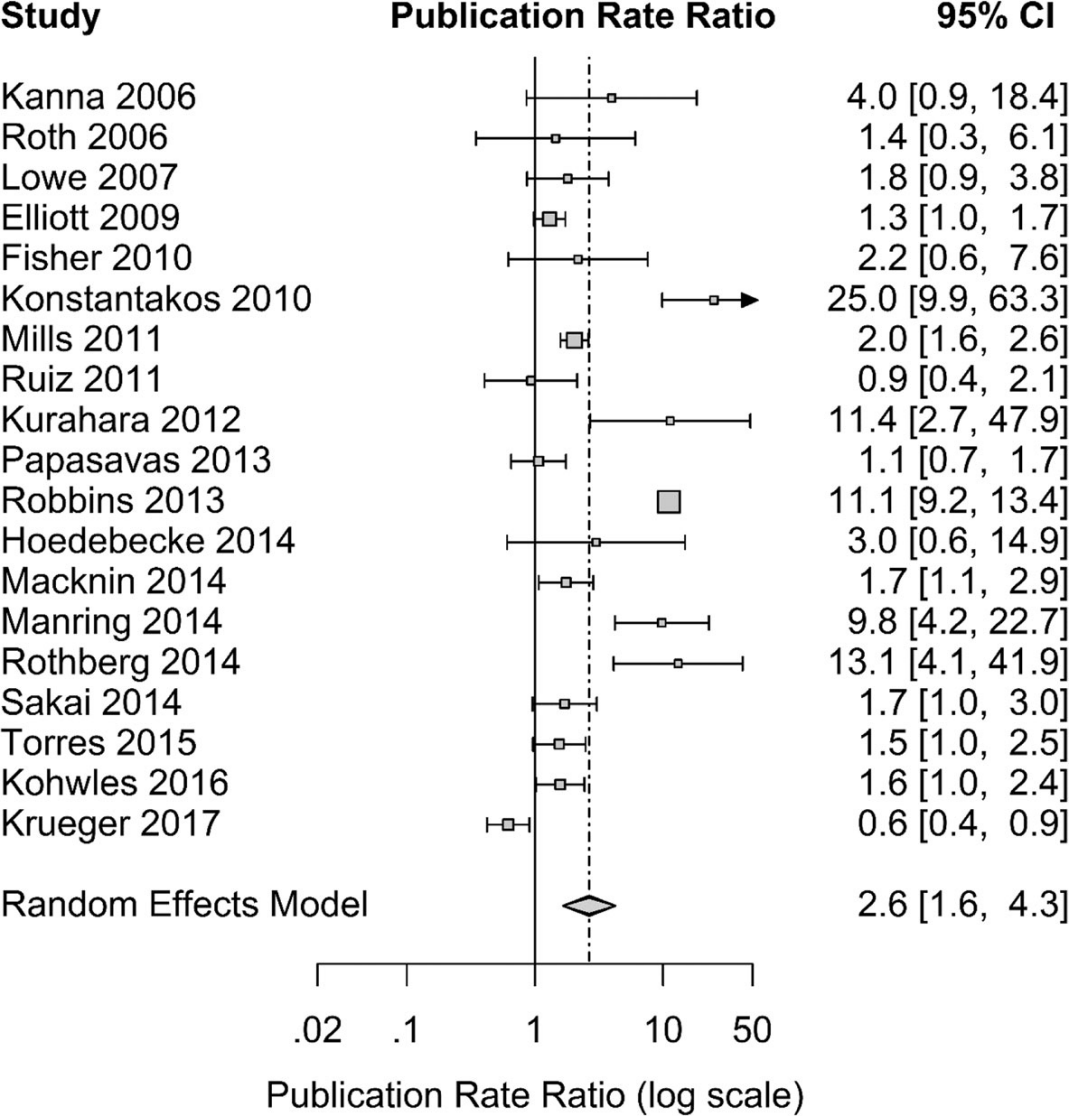
Forest plot for publication rate ratio in a systematic review of the literature on scholarship initiatives in graduate medical education (January 2003–March 2017)

Nineteen of the 32 articles (59%) provided enough detail to calculate a publication rate ratio [3, 7, 21, 23–25, 28–30, 32–34, 36, 38, 40–43, 46]. Two studies reported the percentage of participants who published, but not the overall number of publications; for these studies we assumed one publication per participant [7, 33]. The PRR for these studies ranged from 0.6 to 25, with eight studies having PRR significantly greater than one, indicating that for these programs, the post-intervention publication rates were significantly higher than in the pre-intervention period [21, 23, 24, 28, 40–43]. Overall, the publication rate was significantly higher following implementation of initiatives (*p* < 0.001; Fig. 2); we estimate that the post-initiative publication rate was 2.6 times (95% CI: 1.6 to 4.3 times) the pre-intervention rate, or a 160% increase.

**Fig. 3:**
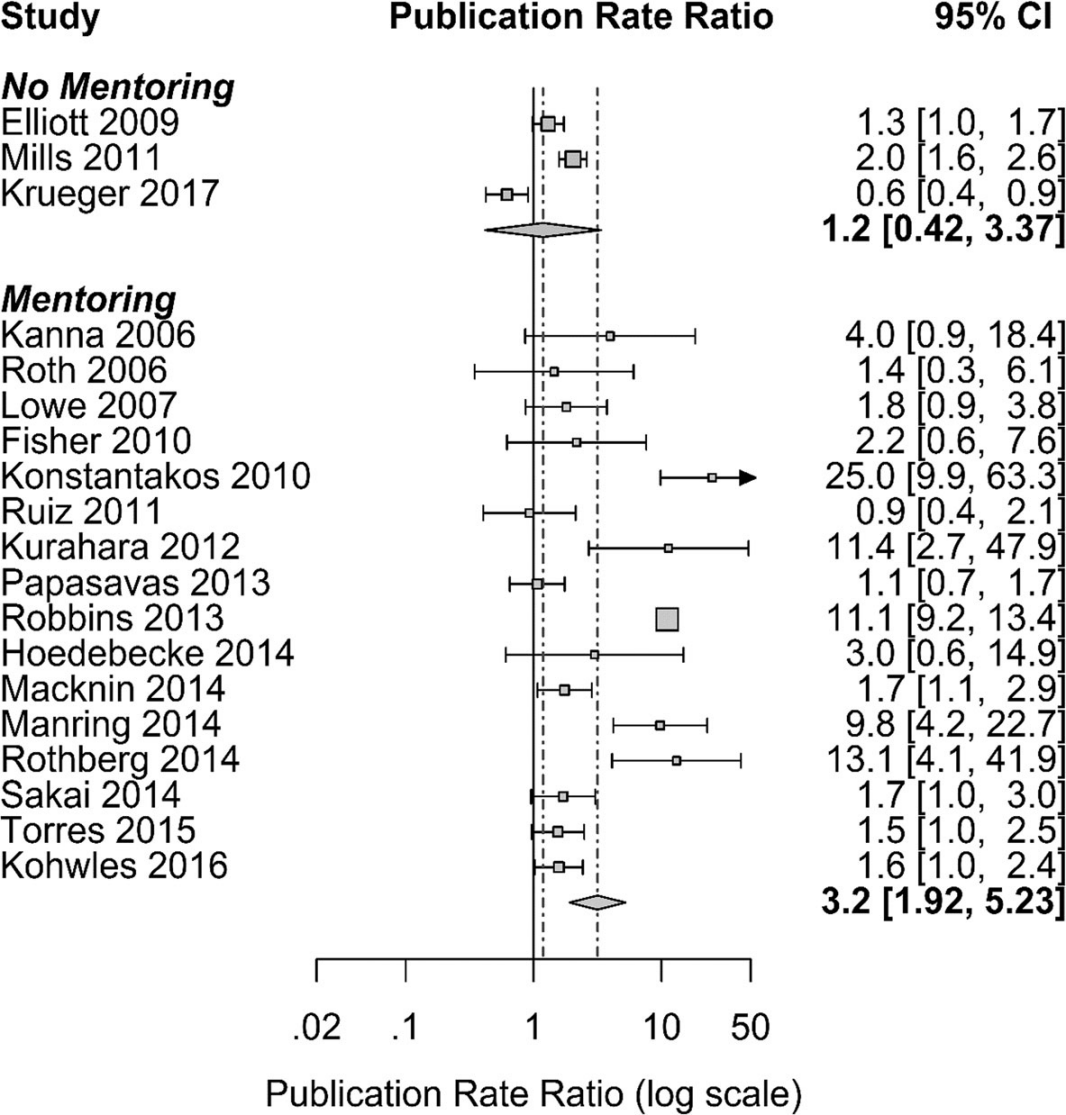
Forest plot comparing publication rate ratio for programs that provided mentors to programs that did not provide mentors in a systematic review of the literature on scholarship initiatives in graduate medical education (January 2003–March 2017)

Of the eight initiatives (mentors, curriculum, protected time, a mandatory component, journal club, grant writing guidance/assistance, funding, and support staff) identified in the included studies, mentoring, curriculum, and protected time were offered most frequently. For this reason, these three initiatives were selected for inclusion in the meta-regression to compare the PRR for programs providing those initiatives to those that did not. Sixteen of the 19 (84%) included programs provided mentors [3, 23–25, 28, 29, 32–34, 36, 38, 40–43, 46]. For programs that provided mentors, the post-initiative publication rate was estimated to be 3.2 times (95% CI: 1.92 to 5.23 times; *p* < 0.001) the pre-initiative publication rate, while the pre- and post-initiative publication rates for programs not providing mentors did not significantly differ (*p* > 0.20) (Fig. 3). However, the difference in the publication rate ratios for these two groups did not reach statistical significance (*p* = 0.10). Programs that provided curriculum (12/19 or 63% of included studies) or protected time (63% of included studies) also did not have significantly higher PRR than programs that did not use these initiatives (*p* > 0.20, for both).

### Publication Bias

The Egger test did not detect any evidence of publication bias (*p* > 0.20). However these results should be interpreted with caution, as it is likely that residency programs that did not see improvements in productivity, or saw a decline in productivity, might be less inclined to publish their data.

### Barriers

We identified 43 barriers that could be organized into 6 major categories (Table 2). The most frequently reported barriers were lack of: time (17/43, 40%), mentoring/oversight (10/43, 23%), and support (6/43, 14%).

**Table 2:** Systematic review of scholarship initiatives in graduate medical education (2003-March 2017): barriers and strategies found in included article abstracts and/or discussions

Barrier Category- 43 Barriers Identified	Frequency^a^
Time (*n* = 17)	
Lack of resident time [17–20, 23, 26, 29, 40, 41]	9 (28.1%)
Lack of time due to clinical responsibility [3, 20, 26, 29, 32]	5 (15.6%)
Lack of curriculum [23, 38, 44]	3 (9.4%)
Mentoring or oversight (*n* = 10)	
Lack of mentoring [17, 19, 23, 29, 35, 41]	6 (18.8%)
Lack of oversight [19, 21, 25, 35]	4 (12.5%)
Funding [20, 29, 31, 32, 40]	5 (15.6%)
Support or training (*n* = 5)	
Lack of support [29, 41]	2 (6.3%)
Lack of training [19, 26, 38]	3 (9.4%)
Lack of Interest [18, 20, 41, 44]	4 (12.5%)
Other [3, 24, 26]	3 (3.1%)
Strategy Category- 117 strategies identified	Frequency
Curriculum (*n* = 22)	
Structured Program [3, 18, 19, 22–25, 27, 28, 31, 32, 35, 36, 39, 42, 43, 46]	17 (53.1%)
Didactic [22, 23, 31, 32, 43]	5 (15.6%)
Mentorship [3, 17, 19, 22–25, 27, 28, 30–32, 34–36, 38, 39, 42, 43]	19 (59.4%)
Infrastructure and Departmental Support (*n* = 16)	
Departmental support [17, 22, 24, 29, 31, 34, 37–39]	9 (28.1%)
Infrastructure [22, 25, 31, 35, 40, 41, 43]	7 (21.9%)
Awareness of Research Opportunities [3, 23, 24, 29]	4 (12.5%)
Collaboration [29, 31, 32]	3 (9.4%)
Protected Time [25, 29, 31, 35, 38–40, 43, 44]	9 (28.1%)
Recognition [3, 7, 24, 35, 39, 43]	6 (18.8%)
Positive Culture [3, 24, 32, 41, 42]	5 (15.6%)
Incentives [20, 34, 45]	3 (9.4%)

^a^Frequencies are percentage of articles that included this barrier or strategy

### Strategies

We identified 117 strategies that could be organized into 9 major categories (Table 2). Of the strategy categories, providing curriculum was the most frequently reported (22/117, 19%), followed by mentorship (19/117, 16%), and infrastructure and departmental support (16/117, 14%).

## Discussion

This systematic review of GME scholarly activities initiatives identified 32 relevant articles published after 2006. All included articles demonstrated improvements in resident productivity in regards to publications or presentations. Unfortunately, many of these articles (66%) failed to specify whether or not their improvements were statistically significant.

Most included articles used multiple interventions (97%), with providing mentors reported most often (88%). Research curricula or protected time were both provided in over half of studies (59%). However, there was wide variability in both curricula and protected time provided.

Programs with curricula included workshops, a lecture series, or research seminars, with much variability in length and content of sessions. The most frequently taught topics were research design (75%) and statistics (69%). The remaining topics were covered by less than half of programs with research curricula. Programs providing protected time varied from a 2-week rotation to a year-long rotation. This wide range in time commitment dedicated to scholarly activities curriculum and protected time to complete projects makes it impossible to make direct comparisons between programs.

A systematic review of research curricula published over a decade ago concluded that “successful educational interventions should incorporate needs assessment, clearly defined learning objectives, and evaluation methods” [8].^,p61^ Despite this call published in 2003, we found little progress made in the inclusion of needs assessment, objectives, and curricular evaluation. Hebert and colleagues [8] found that only 27% of included articles had a needs assessment. In our review, we found that this percent had increased to 37%, which is an improvement but still falls short of ideal. Likewise, we found a lack of curricular evaluation, with only half (53%) providing evaluation data. Similar to our findings of 11%, Hebert et al. [8], found that 12% of studies used an objective pre-post knowledge test.

Our primary outcomes were presentations and publications. Only 2 of the included studies reported statistically significant increases in presentations, while 10 (36%) reported statistically significant increases in publications. All studies with a research curriculum reported increases in presentations and/or publications. However, only 32% of studies with curricula reported statistically significant improvements. This is possibly due to small sample sizes and resulting lack of power to detect differences. In the future, more robust study designs with larger sample sizes are needed to definitively assess the importance of inclusion of a research curriculum.

Of the remaining interventions, funding was reported in a quarter of the studies. However, the amount of funding varied widely across studies. In fact, all initiative interventions varied a great deal across studies, including length of curriculum, amount of protected time and mentoring provided.

We identified nine categories of strategies commonly reported as important (Table 2). Structured research curricula, faculty mentorship, and providing departmental infrastructure and support were the most commonly cited strategies. Strategies that we identified were similar to those identified by Hebert et al. [8] in their 2003 systematic review of residency research curricula. In their article, they described common curriculum components such as educational goals and objectives, lectures, seminars or small groups; role models; and research mentors.

In addition, we identified barriers to research output noted in the reviewed articles. The most frequently cited obstacle when implementing changes to resident research was lack of time, due to clinical responsibility or the importance of educational curriculum besides research. Difficulties in providing clinical research mentors to residents, lack of resident interest in research, departmental funding towards research and challenges in providing adequate training and support were also identified in our review. Hebert et al. [8] identified obstacles encountered based on learner (e.g., resident resistance, lack of motivation), faculty (e.g., resistance, time/intensity demands, lack of motivation) and institutional (e.g., lack of time, financial barriers, lack of critical support staff). Our review yielded much similar results of institutional barriers, yet very few articles mentioned learner or faculty barriers as described by Hebert. This may reflect a change in academic department attitudes towards research.

Hebert and collegues [8] noted that many articles failed to provide descriptions of feasibility, sustainability, or cost. We found 15 (47%) articles had some mention of cost and/or feasibility of their research initiatives. However, many provided vague statements that would yield very little concrete assistance in determining actual cost or feasibility and no article directly addressed sustainability.

It appears that after implementing research initiatives in a residency, a majority of programs saw an increase in resident publications or presentations. However, we were unable to identify a particular intervention that was associated with statistically significant improvements. Whether it was faculty mentor participation, scheduled research instruction, or other initiative, it appears that any departmental dedication to resident research may increase scholarly productivity. It may be that as the overall culture within a residency moves toward supporting resident research and scholarly activity, resident publications and presentations will increase.

### Limitations

As recently noted in an editorial, medical education reviews are difficult to conduct [47]. Many aspects of published medical education research vary, including study design, operational definitions, educational interventions, subjects, sample size, and outcome measures. All of these differences prevent easy aggregation of data [47]. Despite these limitations, we were able to identify 19 (59%) studies with enough detail to include in a meta-analysis of publication rate ratios.

As with any systematic review, the results are limited by the search strategy and methods used. We addressed these issues by developing a detailed protocol with operational definitions and by using multiple trained reviewers throughout the study process. Our search included 3 databases and was conducted by an experienced medical librarian. In addition, the reference sections of all included articles were reviewed for possible additional articles. Although all of these strategies improve the quality of our systematic review, we may have missed some relevant articles.

As with all systematic reviews of the literature, there exists the possibility of a publication bias against negative studies, resulting in few studies published that did not demonstrate improvements. In addition, we were only able to analyze interventions that were themselves published. There likely exist many residency programs throughout the country that have implemented or updated their resident research/scholarly activity initiatives while not explicitly publishing data on the changes and their results.

Although our meta-analysis concluded that the post-initiative publication rate was significantly higher than the pre-initiative publication rate, this result should be interpreted with caution. The design of the studies included in the analysis were varied, and it is possible that changes in the publication rate may have been due to factors outside of the implementation of an initiative. Meta-analysis results can also be sensitive to publication bias, which is likely to be present for this study.

### Recommendations

When implementing or updating a resident research curriculum it is important to consider all aspects of curriculum development, including conducting a needs assessment, developing goals and objectives, and designing a robust mechanism for curriculum assessment. Further, education leadership should consider using freely available, peer-reviewed, online resources, such as MedEdPORTAL. A brief search conducted by our team yielded two teaching resources devoted to research curricula [48, 49] and another two focused on scholarly activities and research mentor resources [50, 51]. There are likely many more such resources available.

It is vital to address barriers to outcomes early and often, to avoid stagnation or poor utilization of valuable resources. In addition, future studies should provide data on cost, feasibility, and sustainability of initiatives used to improve resident scholarly activities.

## Conclusions

While specific interventions designed to improve resident scholarly activity cannot be individually tied to an increase in resident productivity, it appears that a culture of research emphasis is likely the most important factor in leading to improvements in resident research productivity. However, we call for prospective studies that include a power analysis; a control or comparison group; well defined, quantifiable parameters; and high-quality design to identify best practices for future scholarly activity initiatives. Without these studies it remains difficult for residency education leadership to design cost-effective interventions proven to increase resident scholarly activities (e.g., local, regional, and national presentations and peer-reviewed publications).

## Additional files

Additional file 1: GME Scholarship Initiatives Search Strategy Full electronic search strategy for thee databases: PubMed, Embase, and Scopus. (DOCX 17 kb)

## Abbreviations

ACGME: Accreditation Council for Graduate Medical Education
CDN: Canadian dollar
CI: Confidence interval
FTE: Full time equivalent
GME: Graduate medical education
IRB: Institutional Research Board
MERSQI: Medical Education Research Study Quality Instrument
N/A: Not applicable
NS: Non-significant
PGY: Postgraduate year
PRR: Publication rate ratio
USA: United States of America
