# Supplementary material for: Graduate medical education scholarly activities initiatives: a systematic review and meta-analysis
Source: BMC Med Educ. 2018 Dec 22;18:318. doi: 10.1186/s12909-018-1407-8 (PMC6303993; doi:10.1186/s12909-018-1407-8)
Supplement: Supplementary file 1 — GME Scholarship Initiatives Search Strategy Full electronic search strategy for thee databases: PubMed, Embase, and Scopus. (DOCX 17 kb) [file 12909_2018_1407_MOESM1_ESM.docx]

**Search Strategy for Systematic Review of Graduate Medical Education Scholarship Initiatives**

| **Name** | **Date limits** | **Search Date(s)** | **Other Limits** | **Platform** | **Total References** |
| --- | --- | --- | --- | --- | --- |
| PubMed* | 2003-2015 | 10/18/2015 | English language only | NLM | 1485 |
| Embase** | 2003-2015 | 10/26/2015 | English language only | Elsevier | 453 |
| Scopus*** | 2003-2015 | 11/12/2015 | English language only | Elsevier | 1183 |
| On 12/7/2015, conducted same searches to pick up new results since prior searches. | | | | | |
| PubMed* | 10/18/2015-12/7/2015 | 12/7/2015 | English language only | NLM | 16 |
| Embase** | 10/26/2015-12/7/2015 | 12/7/2015 | English language only | Elsevier | 10 |
| Scopus *** | 11/12/2015-12/7/2015 | 12/7/2015 | English language only | Elsevier | 13 |
| On 2/11/2016, conducted same searches to pick up new results since prior searches. | | | | | |
| PubMed* | 12/7/2015-12/31/2015 | 2/11/2016 | English language only | NLM | 42 |
| Embase** | 12/7/2015-12/31/2015 | 2/11/2016 | English language only | Elsevier | 8 |
| Scopus *** | 12/7/2015-12/31/2015 | 2/11/2016 | English language only | Elsevier | 18 |
| On 4/7/2017, conducted same searches to pick up new results since prior searches. | | | | | |
| PubMed* | 1/1/2016 – 4/7/2017 | 4/7/2017 | English language only | NLM | 269 |
| Embase** | 1/1/2016 – 4/7/2017 | 4/7/2017 | English language only | Elsevier | 420 |
| Scopus *** | 1/1/2016 – 4/7/2017 | 4/7/2017 | English language only | Elsevier | 80 |
| Total (with duplicates) |  |  |  |  | 3997 |
| **Total with duplicates removed** |  |  |  |  | **2980** |

* -Set limits to exclude the following publication types: news, newspaper article, letter, or editorial

** -Set limits to exclude the following publication types: conference abstracts, conference papers, conference reviews, editorials, erratum, letters, notes, and short surveys.

*** Set limits to exclude the following publication types: book, business article, book chapter, conference paper, conference review, editorial, erratum, letter, note, and press release

| **Concept #1** | **Controlled Vocabulary** | **Keywords** |
| --- | --- | --- |
| **Research** | PubMed  Research  Research activities  Activities, research  Research activity  Activity, research  Research priorities  Priorities, research  Research priority  Priority, research  Research and development  Development and research  Laboratory research  Research, laboratory  Emtree  Research  Research report | Research project*  Research productivity  Research training  Research team*  Clinical research  Surgical research  Research career  Scholarship  Scholarly activit*  Scholarly productivity  Scientific inquiry  Scientific methodology |

| **Concept #2** | **Controlled Vocabulary** | **Keywords** |
| --- | --- | --- |
| **Curriculum** | PubMed  Curriculum  Curricula  Short-term courses  Course, short-term  Courses, short-term  Short term courses  Short-term courses  Emtree  Curriculum  Education program | Education  Training  Competenc*  Course*  Program* |

| **Concept #3** | **Controlled Vocabulary** | **Keywords** |
| --- | --- | --- |
| **Graduate Medical Education** | PubMed  Education, Medical, Graduate  Education, Graduate Medical  Graduate Medical Education  Medical Education, Graduate  Internship and residency  Residency and internship  Internship  Internships  Medical residencies  Residencies, medical  Residency, medical  Medical residency  Residency  Residencies  House staff  House staffs  Staff, house  Staffs, house  Emtree  Medical Education  Education, medical, graduate  Fellowships and scholarships  Internships and residency  Residency education  Education, residency  Residency training  Resident training  Residential education | Junior doctor*  Postgraduate training  Master of surgery  Physician scientist*  MD PhD  Senior house officer*  Resident*  Fellow*  Fellowship* |

| **Concept #4** | **Controlled Vocabulary** | **Keywords** |
| --- | --- | --- |
| **Osteopathy** | PubMed  Osteopathic medicine  Medicine, osteopathic  Osteopathic manipulative medicine  Manipulative medicine, osteopathic  Medicine, osteopathic manipulative  Emtree  Osteopathic medicine  Manipulation, osteopathic  Medicine, osteopathic  Osteopath  Osteopathic physician  Osteopathic physicians  Osteopathy |  |

**Search Statement**

**1 AND 2 AND (3 OR 4)**
